# A Point in Medical Ethics

**Published:** 1858-10

**Authors:** Samuel Bradbury

**Affiliations:** Oldtown


*A Point in Medical Ethics.* By Samuel Bradbury, M. D., Oldtown.

In the July number of the London Lancet (reprint,) the editor of that distinguished Journal, speaks with sharp and just condemnation of those medical men, who consult with quacks, but thinks that Mr. Fergusson, the celebrated Surgeon of London, acted honorably and properly, in assisting the quack Bell in his "urgent surgical case."

Now I think the Lancet is generaly correct on all points of medical ethics; I think it is one of the most, if not *the* most poweiful and active of all the enemies to error, superstition and quackery, that the world is blest with; but I do believe that in this case the Lancet is wrong; and that Mr. Fergusson did act very imprudently, and that he has set an example which, if followed by the profession generally, will lead to very disastrous results. That Mr. Fergusson's motives in assisting Bell, (I will not disgrace the title of *Dr.* so much as to prefix it to his—Bell's—name) were pure and honest—I will not at present, doubt, not knowing the character of that gentleman: but that he did "*aid* and *abet*" a pretender, indirectly though it may have been, there can be, I think, not a shadow of doubt. The case referred to, stands briefly thus:—Bell, a homœpathic practitioner, takes charge of a man suffering from paralysis of one side of the body. By reason of his taking the case, two highly honorable medical gentlemen, first in attendance on the patient, viz: Dr. Paley and Dr. Philbrick, find it for their own and their profession's honor to decline further service in the case. The patient while under the care of Bell, suffers retention of urine which he (Bell) chooses not to meddle with, but takes Mr. Fergusson with him to perform the necessary operation, which operation, it appears had previously been performed for him (Bell) by a Mr. Jackson. Now the question is, did Mr. Fergusson *do right* in going with Bell and performing the "urgent" surgical operation of passing the catheter?

Mr. Fergusson says himself, in a letter to the Lancet, "I accompanied Dr. Bell to Lincolnshire in an urgent surgical case. I have not seen the patient since. I do not believe in homœpaths. I do not believe in homœpathy. I give no encouragement to homœpaths." All this may be true, and probably is; but can we not often *aid* people without *consulting* with them? Did not Mr. Fergusson encourage Bell in his infamous practice, by accompanying him to Lincolnshire, and helping him out of his urgent difficulty?

The editor of the Lancet upholds Mr. Fergusson in his conduct, and in the July number of the Lancet, (reprint,) says, "we agree with him (Mr. Fergusson,) that when a surgeon is called upon for assistance in an urgent case, he is bound to give it *pro hoc vice,* even if he knew a charlatan to be in attendance."

The rule the Lancet has laid down, by which to govern physicians, I believe is dangerous, and should not be followed for the following reasons—viz: because assistance given in "urgent" cases, while under the care of quacks, tends, indirectly, but no less surely, to foster and keep alive a rapacious and vile herd of pretenders, by giving them "aid and comfort." We should not "do evil that good may come." We should not encourage error, even if by so doing, we can save a patient's life; for in saving one life, we become the indirect instruments of a thousand deaths.

How precious is *truth* to everybody—especially to the honorable physician. He who trifles with it, is sure, sooner or later to get roughly handled; he cannot escape. Give "aid and comfort" to a quack to-day, and this does not end the matter, as Mr. Fergusson may suppose, when he says as above quoted—"I have not seen the patient since;" no, for Bell had got all the aid he wanted from him. To-morrow when the case again becomes "urgent," he can again call Mr. Fergusson to his "urgent" surgical case; and if he is absent, he can take with him the kind-hearted editor of the Lancet, who cannot refuse to assist in so "urgent" a case.

See where the Lancet's rule will carry us. If we cannot refuse to assist a quack in his "urgent surgical case," how can we refuse to aid him in his "urgent *medical* case;"* for there are many cases in the practice of medicine proper, which require *immediate* attention; and so the *physician* must be brought to the aid of error in any "urgent *medical* case," provided some quack sees fit to make a tool and a "cat's paw" of him.

It is in "urgent" cases, both medical and surgical, that quacks and the public generally, should be made to feel, not only the value and the power of the truth, but the inflexible justice of it also; and then pretenders, having no foothold, will fall by their own shame, and the scorn of the public.

In my humble opinion, the duty of a physician or surgeon when called to an urgent case, or one that is not urgent, is very plain and well defined; *he should refuse to have anything to do with a patient while in the hands of a charlatan;* as assistance on his part becomes injurious to our profession and highly injurious to the public. If a patient or his friends will not dismiss a quack from his rapacious hold, the patient must suffer the consequences. His or their folly must prove the punishment, which shall follow. We will not mix up truth and honor with pretence and villainy. Meantime we should give the public on every occasion to understand by our words and by our acts,

* In fact the Lancet makes no distinction between the two.

the vast difference between science and humbug. Let us show them, (what they are profoundly ignorant of) *the true position of medical science;* how it is advancing daily towards perfection by the laborious efforts of the medical profession; and how it embraces, as it advances, everything within its reach that is found in any way to aid us in relieving the sufferings of mankind. Let us show the public that medica men have *honor* as well as science; that a gross insult offered them shall not go unrebuked; that pretenders and triflers with human life shall have no place beside honorable men; and that ignorance in treating disease must ever be fatal to the welfare of the patient.—*Maine Med. & Surg. Reporter.*

OLDTOWN, August, 1858.

